# Hygiene in University Education

**Published:** 1894-08-18

**Authors:** John S. Billings

**Affiliations:** Deputy Surgeon-General U.S. Army. *Abstract of an Address given to the University Extension Classes, Oxford, England*


### HYGIENE IN UNIVERSITY EDUCATION.

By John S. Billings, M.D., D.C.L., Oxon., Deputy Surgeon-General U.S. Army.

*Abstract of an Address given to the University Extension Classes, Oxford, England.*

The division and specialisation of labour, which are characteristic of modern civilisation, are applied in educational affairs, as well as in the supply of clothing, food, and habitations for the people. Your beds and houses, bread and shoes, are such as they are, and not such as men had when the first students gathered at Oxford—and it is within your power to obtain, as necessities of your daily life, conditions which the Tudor kings could not command as luxuries—because a few men have discovered methods of controlling and using the forces of wind, heat, gravitation, and electricity; because a larger number of men have applied their capital and brains to bringing these methods into use in collecting material, and in manufacturing and distributing the products; and because a multitude of other men have given bodily labour, for daily wages, to carry out the plans of their employers, each labourer doing but a very limited kind of work. In like manner the fact that you are about to receive practical, useful information as to the laws of life and death, the causes of certain unnecessary disease and suffering, and the means whereby these may be averted or prevented, depends on the fact that about half a dozen men have spent years in making observations and experiments, and devising methods for the determination of the nature of these causes, that an hundred other men have made it their life work to apply these methods of investigation to the details of particular diseases, and that a much larger number of men have studied the results thus obtained and are engaged in their practical application and in teaching others how to use them. All of these agencies are necessary to obtain the end desired, but they are not all equally important, because some of them are more easily obtained than others, yet we need them all.

### What a University Education Should Include.

A University education in hygiene should not only include information as to the nature and relative importance of the principal known causes of disease and death, immediate and remote, but also as to the cost of the means of partially or entirely doing away with these causes, as compared with the value of the results which may be thus obtained. It is not always worth while to be careful to avoid a danger; one may pay too much for a life insurance; there are times when one should go to battle, or meet a pestilence, or live in an unhealthy locality, when it is merely one's own life that is at stake; and there are also occasions when one should take the responsibility of leaving, or putting, in danger the lives of a few to preserve the welfare of the many. The importance and value of long life is always taken for granted in discourses upon hygiene; but if it were in your power to prolong the life of every one in England to one hundred years, would you do it? If you were offered as a gift that you should not die until you were a hundred years old, would you accept it without conditions? Why do we expend time and money to secure at public expense the preservation of the health and prolongation of the lives of congenial idiots, of the hopelessly insane, of hereditary and confirmed criminals? If there is truth in the doctrine of the survival of the fittest, why do we intefere in the struggle for existence, and try to shift the inevitable penalties for the violation of Nature's laws upon those who have not violated them? Was Carlyle right when he said, "Let wastefulness, idleness, and improvidence take the fate which God has appointed them, that their opposites may have a chance for their fate. He that will not work according to his faculty, let him perish according to his necessity?" When you come to consider these and similar questions, you will see that the problems of hygiene are not m[illegible]y mechanical, chemical, bacteriological, and medical; they embrace, also, sociological, political, and ethical considerations, and the world needs some "all-round men" to study them. Such men the great Universities should endeavour to equip for this work.

### Epidemics and Destiny.

The part which epidemic diseases have played in shaping the destinies of cities and of nations has rarely been investigated by historians. Even the two greatest epidemics of which we have any record, the Justinian plague and the Black Death, have received little more than a brief mention in the leading English histories; and yet each of these not only changed the map of Europe, but also exercised a profound influence upon the social customs and the religion of the people, an influence which has affected the new world as well as the old. The influence of epidemics on the progress of nations and communities is by no means merely a matter of ancient history. Yellow fever has, to a considerable extent, shaped the present commercial, social, and political condition of the West Indies and Central America, of New Orleans and Rio de Janeiro. Cholera was the immediate cause of modern hygiene: its ravages have been closely connected with certain religious observances, and its prevention is still a problem which must be solved by British statesmen; while the future of the continent of Africa depends quite as much upon progress in hygiene as upon political considerations. The International Conference at Paris, only four months ago, shows that questions of hygiene play an important part in matters of international comity, and the International Congress of Hygiene and Demography which last met in London, and is to meet again next September in Buda Pesth, is exercising an increasing influence upon public health legislation in all countries, an influence none the less powerful because it is persuasive and not compulsory.

### Many Things that are yet Unknown.

Your teachers in hygiene can tell you what is known with regard to the causes of disease and methods of prevention, and that is much; but I am more concerned in this lecture to call your attention to the many things that are yet unknown, and yet which probably might be known with a little work, in matters affecting practical hygiene. Let us take a few concrete examples. What is the cause of measles? How is it communicated? What is the probability that the disease can be communicated by a person after the eruption has fully developed and the diagnosis is certain? We do not know. We know that the disease is most contagious during the three or four days preceding, and during the first day of the eruption, and that attempts to prevent the spread of the disease in a family by isolating the patient as soon as the eruption appears, rarely succeed, while the contrary is the case in scarlet fever and in small-pox. We suppose that the medium of contagion is the secretion from the upper air-passages, which is scattered in spray by the coughing and sneezing of the patient. Does the susceptibility of measles diminish with advancing years, as does that of scarlet fever? We do not know; but the experience of the Faroe Islanders, and of the American recruits brought together at the beginning of our last war, indicates that while it is most fatal in children under five years of age, it may be quite as contagious and nearly as fatal among adults as among children. Now all these are questions to be considered in deciding as to whether notification and isolation of cases of measles are to be enforced in any particular locality. We have fairly good statistical evidence that the evil effects of scarlet fever, diphtheria, and small-pox have been diminished by compulsory notification and isola-

HYGIENE IN UNIVERSITY EDUCATION—*continued.*

tion; but I know of no such evidence with regard to measles. Measles appears to have caused at least twice as great a mortality in the large cities of England and Continental Europe during the last five years as it has in the United States; but why this is so I cannot tell. The death-rate from measles is small as compared with that from accidents, or from many other forms of disease; but its evil effects are not to be measured by its death-rate alone.

**The Perils of Infant Life.—Measles.**

Dr. Hugh R. Jones,* in an interesting paper on the perils of infant life, presented to the Royal Statistical Society in December last, says: "The danger of measles has been very greatly underrated. There is no doubt but that the tuberculous diseases of childhood, whether local, as lupus and enlarged glands, or whether general and fatal, as pulmonary phthisis, are often rightly traced and attributed to an attack of measles." There is no definite evidence upon this point, which is an important one. In the United States a considerable proportion of cases of feeble-mindedness, deaf-mutism, and blindness are attributed to measles, sometimes erroneously no doubt; but we must certainly consider this disease as of sufficient importance to warrant special precautions to prevent its evil consequences. I doubt, however, whether it is worth while to expend much public money, or to interfere with home life, in order to prevent it among children over five years of age; and the true solution of the problem will probably be the production of a mild foım of the disease in children, and thus making them immune for the rest of their lives. This is practically what has been done for centuries in China with regard to this disease; and, when I was a boy, I have known people to deliberately expose their children to, the infection of a mild case of measles in warm weather, in order "to get the disease through the family without risk," as they said. Something of the same sort used to be done by northern men settling in New Orleans, in purposely exposing themselves to yellow fever when it existed in a mild type, in order that they might beeome "acclimated" as the phrase went.

**Typhoid Fever.**

Let us next take typhoid fever. Typical cases of this disease are caused by a specific bacillus which gains entrance to the body through food or drink contaminated with the excreta of persons affected with this disease. Epidemics are due to contaminated water or milk. The stools of a typhoid case can be cheaply and easily disinfected so as to destroy the specific bacillus, and I presume that many persons think that we know all that need be known to stamp out typhoid fever or, as the English registrars call it, enteric fever. In the United States, however, we have many cases of mild continued fever, lasting from twenty to thirty days, which we presume to be typhoid fever, but which are certainly not typical, and which shade into continued malarial forms of fever in a very puzzling way. There are also several known varieties of the typhoid bacillus; and it is not quite certain that one or more of these varieties do not exist at times in the intestinal tract without producing a specific fever in the person bearing them. Can the specific, active typhoid bacillus be developed from some of these, under certain certain circumstances, in privy vaults, cesspools, or sewage? Is the typhoid bacillus carried in currents of sewer air? Are not all the cases in which it has been supposed to have been thus carried to infect men, more easily explained in other ways, as, for example, by the supposition that it has been conveyed to articles of food or drink through the agency of flies and other insects? What degree and duration of immunity from subsequent attacks does an attack of typhoid fever confer on a man? Is there any difference between the immunity conferred by a mild, and that conferred by a severe, attack? Are there attenuated varieties of the typhoid organism? Can these be developed into more dangerous forms under certain conditions? Can they be used to produce immunity?

* "Journal of the Royal Statistical Society," lvii., 1894, p. 15.

**Tuberculosis and Consumption.**

All of these are as yet unsolved, yet probably solvable problems; and there are similar ones connected with each of the known and unknown pathogenic micro-organisms upon which bacteriologists in various parts of the world are working. Tuberculosis causes, in England and the United States, much more suffering and loss of life than any other single form of disease; more than cholera, yellow fever, small-pox, scarlet fever, typhoid fever, measles, and diphtheria put together. Its immediate or efficient cause, the bacillus, has been carefully studied; and some of the modes of its transmission, through dried sputa, milk, &c., are fairly understood. To what extent heredity is a factor in its transmission we do not know. Quite recently, in many places in the United States, it has been proposed to limit its ravages by means of compulsory notification, isolation of the sick, disinfection of their surroundings, and official supervision of milk and meat supplies; and, in a few places, some of these methods are being tried. The results of these experiments will be watched with great interest; they are good examples of the steadily increasing tendency to interfere with the liberty of the individual for the supposed benefit of the community. The death-rate from consumption has been diminishing for the last twenty years in the eastern portion of the United States, and in most countries from which we have reliable statistics. Part of this decrease in the death-rate is probably due to improvements in food supply and in general sanitation, involving greater cleanliness and more exposure to sunlight, which last is one of the most powerful agencies in destroying the infection of tuberculous sputa scattered in the streets; a part of it, during the last ten years, is due to the education of the people as to the necessity of promptly disinfecting the sputa of tuberculous patients; and a part is probably due to a gradual increase in the proportion of persons who are immune against small doses of the tubercle bacillus, which increase is brought about by the law of natural selection. I shall refer presently to this immunity as connected with race, in relation to consumption, and also to pneumonia, another very fatal disease due to micro-organisms.

**Foul Air and Ventilation.**

Now let us take an entirely different field of hygiene, namely, the effects produced on the health and life of men by inhaling air which has recently been expired by themselves or other men, and which also contains the exhalations from their skins. It is generally accepted as a truism that air thus vitiated is dangerous to health, and that ventilation of living-rooms, bedrooms, barracks, school-rooms, &c., is an important sanitary measure. Forty or fifty years ago the danger was supposed to be due to carbonic acid, and the popular ideas on the subject are contained in the school-boy's composition on "Breath." He said: "Breath is made of air. We breathe always with our lungs, and sometimes with our livers, except at night, when our breath keeps life going through our noses while we are asleep. If it wasn't for our breath, we should die whenever we slept. Boys that stay in a room all day should not breathe; they should wait till they get outdoors. For a lot of boys staying in a room make carbonicide; and carbonicide is more poisonous than mad dogs, though not just the same way." For the last thirty years the danger has generally been attributed to exhaled organic matter of unknown composition, but with poisonous properties, while within the last two years several experimenters have announced that the organic matter is not dangerous, and several

**HYGIENE IN UNIVERSITY EDUCATION**—*continued*

researches are now going on to settle this important question. Now, what are the effects upon men of exposing them for eight or ten hours a day to air rendered impure by their own exhalations, and to what particular change in the air are these effects due?

**Inhabited Rooms and Disease.**

From the days of the English Barrack Commission down to about ten years ago the answer was, that such air produced consumption and other pulmonary diseases. Now that we know that consumption and croupous pneumonia are produced by specific bacteria, the question is whether, if these bacteria are destroyed and kept out of inhabited rooms, which can be done to a very great extent, the foul air of an ordinary room will produce disease, and if so, what sort of disease. We do not know; and therefore we cannot produce scientific demonstrative evidence to convince architects and engineers that each man ought to have a certain stated supply of fresh air per minute or per hour, and that it is worth the cost of special apparatus to ensure this. Much the same may be said about the supposed injurious effects of sewer air, since we have no accurate knowledge or satisfactory evidence with regard to these effects. There are two or three times as much sickness, and two or three times as many deaths, in crowded tenement-houses as in single dwellings of the better class, and these tenement-houses are unventilated or badly ventilated; but how much has the bad ventilation, and how much the general want of cleanliness, insufficient and improper food, alcoholism, and vices of various kinds found among the tenement-house population, to do with this matter? And what are we to say about the low death-rate in tenement-houses occupied by Jews, except that race seems to exercise a powerful influence in producing this result. In the course of lectures on hygiene which you are to have, you will no doubt hear much about the pathogenic bacteria, as causes of tuberculosis, typhoid fever, diphtheria, pneumonia, wound infections, cholera, &c., since an understanding of their nature, habits, and modes and conditions of growth is essential to the proper dealing with contagious and infectious diseases of all kinds, even those for which no specific micro-organisms have yet been discovered. But there are other things besides micro-organisms to be considered in matters of personal, national, or international health, because even for those diseases of which they are the immediate cause, there are remote causes of great importance, such as heredity, poverty, ignorance, climatic conditions, overcrowding, alcoholism, occupation, and other things which destroy many without the aid of specific bacteria, and do much to make possible the destructive work of such organisms.

**The Relation of Race to Health.**

During the twenty years ending in 1890, the annual death-rate in Austria was 30·6; in Italy, 28·6; in Prussia, 25·6; in France, 22·8; in Belgium, 21·4; in England and Wales, 20·3; in Ireland, 18; in the United States, 18; and in weden, 17·6 per 1,000 of living population. Such figures, owever, prove very little, for in comparing the vital statis- ics of different countries and nations, either as to general eath-rates or death-rates due to particular diseases, it is npossible to say what proportion of the difference observed due to differences in climate and food, and what to race eculiarities. This difficulty can be in part avoided by xaming the vital statistics of different races living under the same conditions as to climate, &c.; and the data coming from certain portions of the United States, which is now the great mixing-ground of races, are especially valuable in this respect. In 1890 the city of New York contained about 335,000 white persons whose mothers were born in America, and 25,000 coloured; 400,000 whose mothers were born in Germany; 400,000 whose mothers were born in Ireland; 120,000 Russian and Polish Jews; 55,000 Englishmen; and 54,000 Italians. You will see that it had a larger Irish population than any city in Ireland, and that but three cities in Germany exceed it in the number of German population. Taking the deaths among persons fifteen years old and upwards for the six years ending May 31st, 1890, we find that the annual death-rate per 1,000 of population in these different races are as follows: Irish, 28; coloured, 23·6; English, 20·8; Germans, 17·0; Americans, 16·0; Italians, 12·3; Russian and Polish Jews, 6·2. The low death-rate of the Jews has been noted in Germany and France also. In New York city they occupy some of the most crowded tenement-house districts. A considerable number of those reported as Germans were Jews with a low death-rate; and if these could be separated, the death-rate of the Germans would probably be over 19 per 1,000. These are general death-rates only. Let us see what the figures are for certain causes of death. The annual death-rates for consumption were, for each 100,000 persons: Coloured, 774; Irish, 646; Germans, 329; Americans, 205; Russian and Polish Jews, 98. For pneumonia, the death-rates per 100,000 persons of all ages were: Italians, 456; coloured, 390; Irish, 344; American whites, 273; English, 269; Germans, 214; Russian and Polish Jews, 170.

**Death Rates and Race.**

I will not weary you with further details of figures, which those of you who are specially interested in the subject will find in the reports of the Vital Statistics of the Eleventh United States Census, but will merely say that the corresponding data from Boston, Philadelphia, Baltimore, Washington, and from the new England States as a whole, taken with those from New York State and New York City, and with those derived from a special investigation of over 10,000 Jewish families, including over 50,000 persons, lead to the following conclusions as being probable for the United States: 1. The coloured race is shorter-lived than the white, and has a very high infantile death-rate; it is specially liable to tuberculosis and pneumonia, and less liable than the white race to malaria, yellow fever, and cancer. 2. The Irish race has a rather low death-rate among its young children, but a very high one among adults, due to a considerable extent to the effects of tuberculosis, pneumonia, and alcoholism. 3. The Germans appear to be particularly liable to disorders of the digestive organs and to cancer. 4. The Jews have a low death-rate and a more than average longevity; they are less affected than other races by consumption, pneumonia, and alcoholism, but are especially liable to diabetes, locomotor ataxy, and certain other diseases of the nervous system.

**The Effects of Heredity.**

The effects of heredity upon liability to diseases and death appear to be due in part to difference in structure and composition of the tissues and fluids of the body, but to a much greater extent to differences in place and mode of life connected with relative poverty and ignorance. The bacillus of tubercle and the micrococcus of pneumonia are affected as to their growth and development by heredity peculiarities of structure of men; in other words, just as many individuals are more or less immune against these organisms under ordinary circumstances, so, also, are certain families and races; and the same is true as regards a large number of the contagious diseases, as well as some which are not contagious so far as we now know, such as cancer, diabetes, hystero-epilepsy, and scleroses of the brain and spinal cord. Can this relative immunity be developed or increased in an individual or a family by artificial means, or by regulations of the habits and modes of life? And, if this can be done, what effect will the production of immunity against one micro-organism or disease have upon the effects of another micro-organism or cause of disease? If

HYGIENE IN UNIVERSITY EDUCATION—*continued.*

we can produce a branch of the Irish race which will be as immune against the bacillus of tuberculosis as are the Jews, will that race be specially liable to diabetes or cancer? As all men must die, the effect of stamping out one particular form of disease must be to increase the number of deaths from other causes, and in this sense it is true that vaccination has increased the number of deaths from accidents, from suicide, and from consumption, because it has preserved children from small-pox to die at a later period from these other causes; but we have not a particle of evidence that the immunity against small-pox produced by vaccination is the cause of, or is accompanied by, a less immunity against some other disease. It should be remembered that for all diseases of which one attack produces immunity, the tendency is, in the course of many generations, to make the whole population subjected to such an influence immune.

### Work for the Universities.

Perhaps some of you may think that such questions as I have suggested are purely theoretical, unanswerable, and, therefore, of no practical interest; but it is not so. Some of them, if not all, can be answered, and they ought to be answered. To do this, new lines of investigation must be opened—partly experimental, in well-appointed laboratories; partly by collective investigations by medical men and in hospitals; partly new methods of statistical research based on disease, as well as on death registration and the census. And the universities should train some men to understand the importance of this work—the men who are to become legislators and heads of departments, and some other men who can do the work if means and opportunities are afforded them. Observe that I say "should train some men," not "should train all their students." Of every hundred students at a university, not more than half a-dozen can be developed into original investigators and thinkers, and probably not more than one in a thousand can ever be induced to devote himself seriously to such problems as I have indicated, because, as a rule, the only reward that can be expected is the satisfaction derived from the work itself. Such work requires much time, great skill and patience, and the opportunities which only a well-equipped laboratory or an official position can furnish. The universities cannot produce the men qualified to do such work—"God alone can make an artist or a man of science"; but they can take care of such men when they appear, and can give them opportunity and encouragement.

### The Importance of Experimental Pathology.

If we accept the ordinary definition of hygiene as being the art of preserving and improving health, it is not so much this technological matter that a university education should include as the scientific foundations upon which the art rests. It does not appear desirable that every university man should know the proper gradient of a sewer, the best form of traps and house-drainage fixtures, the peculiarities of patent ventilators, or the proper construction of a hospital for contagious diseases, any more than he should know how to treat a case of pneumonia, or how to draw up a conveyance of a piece of real estate; but he should know where to go for accurate and reliable information and advice on these subjects. It is now generally admitted that biology is a branch of science for which universities should provide the means of increase and of diffusion of knowledge; but it has not yet been generally understood that morphology and physiology, as ordinarily provided for in university work, do not cover the most important part of biology, that part to which they are merely necessary preliminaries, that part which is the main reason for their existence, and without which they rest on narrow scientific foundations, namely, pathology. We cannot be said to understand the structure and functions of an organ until we know what these are in its abnormal as well as in its normal condition. It is to experimental pathology in its broadest sense, including not only the study of the lesions specially produced for the purpose, but also the study of the lesions produced in man and animals by disease, each case being one of nature's experiments, that we must look for the most valuable explanations of peculiarities of structure and function, for explanations of the mode of action of physical, chemical, and vital agencies in the production of disease, for means to counteract the abnormal conditions and actions of organs and tissues; in short, for the scientific foundations of hygiene.

### A Department of Pathology for Original Research.

It appears to me that at the present time the majority of the English and American universities are in urgent need of a department of pathology properly equipped for original research and for teaching, not as a mere technological matter, or as merely a branch of the medical department, but as a department of general biology; and the organisation of such a department should precede or accompany the organisation of a department for the promotion of scientific hygiene.

### A Seasonable Protest Against Impractical Teachers.

While I lay stress on the promotion of original research in problems of hygiene as one of the most important functions of a great university of the present day, it is by no means its only one. It is also to train teachers, men who can explain to others what is really known on these subjects, and the consequences thereof, in a fashion which will command attention, interest, and belief. It is knowledge of the truth about these things which frees a man from much unnecessary pain, causeless fears, and useless labour, and from being swindled in a thousand ways; and I presume that this university extension course in hygiene is intended to furnish knowledge, and also to create a thirst for more. To one who has had little practical experience in sanitary matters, the importance of educating the people on this subject is not sufficiently apparent, although he may admit it as a matter of theory. He is too apt to suppose that the people at large can be made healthy by regulations enforced by officials employed for the purpose. He wants laws to suppress tuberculosis, puerperal fever, cerebro-spinal meningitis, &c., by means of enforced notification, isolation, and disinfection. He would have a certain hourly supply of fresh air furnished by law to every sleeping-room; would compel every one to take a bath every day, or at least once a week; would have all food officially inspected, and prohibit the use of alcoholic drinks and tobacco to men under fifty years of age; would not allow the sale of corsets, or of shoes which do not conform to the natural shape of the foot; would regulate the hours of study and of exercise for school children, and have inspectors examine their toys and picture-books for dangerous colours; in short, it is difficult to foretell what he would not do by legal process, if he could, to prevent what he thinks is injurious to life and health, and as a general rule he will find plenty of people who will passively assent to such propositions. Perhaps, on the same ground, may be advocated the refusal of permission to marry unless both parties to the contract have been approved by skilled inspectors, or the official assignment of persons to particular occupations best suited to their health. It is possible that, by these and other regulations which will no doubt occur to you, a healthy community might be produced in time, provided that great care was taken to prevent anyone from getting away. And no doubt the civilised part of the world is at present tending to increasing interference with the liberty of the individual for the real or supposed benefit of the community; but attempts to hasten this progress in advance of the education of the community, or without due consideration of the manifold social, commercial, and professional, as well as the sanitary, interests involved, are not likely to produce good results; on the

HYGIENE IN UNIVERSITY EDUCATION—*continued.*

contrary, it is probable that their remote effects may be the injury of the very cause which their enthusiastic advocates are trying to promote. You cannot legislate a new layer of cortical gray matter into, or a cirrhosed liver out of, a man. The scientific foundations of practical hygiene have been immensely broadened and strengthened within these thirty years; and within the same period, in most civilised countries, the death-rates have been lowered, the average duration of life increased, and life, while yet it endures, has been freed from some of its pains and terrors. But the community of perfectly healthy men and women does not yet exist, nor is it possible that such a community, if found, could continue to exist indefinitely if the present rate of increase of population of the earth shall continue.

### Diminution in Birth and Death Rates.

During recent years the birth-rate has been diminishing in proportion to population in most civilized countries, as will be seen by the following table.

Birth-Rates per 1,000 Population.

| Country. | 1880. | 1890. |
|---|---|---|
| United States | 36·0 | 30·7 |
| England and Wales | 34·2 | 30·2 |
| Scotland | 33·6 | 30·3 |
| Ireland | 24·7 | 22·3 |
| France | 24·5 | 21·8 |
| Belgium | 31·1 | 28·7 |
| German Empire | 37·6 | 35·7 |
| Austria | 38·0 | 36·7 |
| Switzerland | 29·6 | 26·6 |
| Denmark | 31·8 | 30·6 |
| Norway | 30·7 | 30·0 |
| Netherlands | 35·5 | 32·9 |

This diminution of the birth-rate began in most countries in 1876, and is a matter of considerable importance in the sanitary as well as in the sociological problems of the future, for it must be given due consideration in making plans for regulating by law the health, the labour, and the lives of men. What are the relations between diminishing death-rates, diminishing birth-rates, and diminishing marriage-rates? How much of the lowering of the death-rate in recent years is due to public sanitation? How much to improvements in medicine and surgery? How much to increasing immunity of the great mass of the people to certain forms of disease? How much to better and cheaper food supplies? Is it more probable that twenty years hence the death-rates will be lower, or that they will be higher than they now are?

### What of the Future?

It seems to me that a great university which is worthy of its name should provide for the training and equipment of a few men to consider these and similar questions, and for the training of many men who are to be the future legislators for, and advisers of, the people, in such fashion that they can appreciate, and make practical applications of, the conclusions to which the special students shall arrive. Just at present the practical problems of public hygiene relate mainly to masses of men, to cities, bound together by the iron lines of the railway and the telegraph; but as the coal supply diminishes, the cities will begin to diminish also, unless our engineers will give us some new means of storing the forces of the sun's rays, of the winds, or of the tides, and when that happens, sanitary questions will become of the first importance for all countries. "As of the leaves on a thick tree, some fall and some grow; so is the generation of flesh and blood, one cometh to an end and another is born." Whatever happens, we must all continue to live in the shadow of the hawk's wing, as our forefathers have done, since each has but a certain span of life, which he cannot lengthen, although he may easily shorten it. He can, however, learn not to be afraid of this shadow—learn to look up and not down, to look out and not in; and one of the best means of doing this is to devote time and thought and labour to the helping of others to help themselves, which is the essence of public hygiene, as it is of true charity and of all real human progress.

